# Motivators and Motivations to Persist With Online Psychological Interventions: A Qualitative Study of Treatment Completers

**DOI:** 10.2196/jmir.2100

**Published:** 2012-06-22

**Authors:** Liesje Donkin, Nick Glozier

**Affiliations:** ^1^Brain and Mind InstituteThe University of SydneyCamperdownAustralia; ^2^Disciplines of Psychiatry and Sleep MedicineSydney Medical SchoolThe University of SydneyCamperdownAustralia

**Keywords:** Adherence, persistence, eHealth, online interventions, motivation, barriers

## Abstract

**Background:**

Many users of Internet interventions do not persist with the full treatment program. As persistence may influence outcomes of such interventions, being able to maximize persistence is vital. However, while studies have begun to explore the predictors of dropout in Internet interventions, few have explored reasons why users persist with the programs, which may not just be the converse of the reasons for dropout.

**Objective:**

To answer the question of what influences persistence with online interventions.

**Methods:**

We interviewed participants in the Cardiovascular Risk E-couch Depression Outcome (CREDO), a trial evaluating the efficacy of an eHealth intervention (e-couch) in treating depressive symptoms in those with comorbid depression and cardiovascular risk factors. Interviews were semistructured in nature and were analyzed using a grounded theory approach. Interview numbers were curtailed (n = 12) after theoretical saturation.

**Results:**

All participants reported substantial barriers to completing the program including time constraints, competing priorities, anxiety about spending time on the computer, and perception of limited worth of the intervention. Participants who persisted with the trial reported intrinsic motivations such as personal values about task completion and sense of control, and recognized external motivators that aided the development of habits and identified personal benefits attributable to the program.

**Conclusions:**

Online interventions may benefit from content that enhances the intrinsic motivations such as a having sense of control and being able to identify with the program, and by increasing the relative value of the program in order to enhance persistence. Persistence within a trial setting appears modifiable through explicit messages regarding supporting others. In terms of motivators, the use of a hook to engage participants who are starting the intervention due to curiosity and the use of reminder systems to prompt participants may also improve persistence. The worth of such additions should be evaluated using adherence and outcomes metrics.

**Trial Registration:**

Australian New Zealand Clinical Trials Registry (ANZCTR): ACTRN12610000085077; http://www.anzctr.org.au/ACTRN12610000085077.aspx (Archived by WebCite at http://www.webcitation.org/68MtyPO3w)

## Introduction

As acceptability of the Internet and Web-based programs increases [1-5], so too does the volume of Web-based interventions for mental health and health behaviors [6-10]. Such interventions are not necessarily standalone interventions, but may be used in a stepped-care model [11,12], as an adjunct to face-to-face therapy to enhance the availability of access to mental health services [4,13], or as an intervention supported by a health care worker [14,15].

Increasing interest in online interventions or e-therapy has largely been driven by the belief that delivering interventions over the Internet overcomes some of the barriers of traditional face-to-face therapy. Such issues include geographical access, time restrictions, cost of accessing services, and reluctance to seek help [16-18]. The Internet as a mode of delivery may overcome these barriers through being available where and when the client needs the intervention [1], and by promoting help seeking and disclosure through anonymity [19-21]. Additionally, some studies have found that online interventions are less affected by behavioral avoidance of session attendance [22], are more cost effective [23], and may reach people whose symptoms are more severe than the symptoms of those who present to a clinic [1]. Importantly, some research participants have also indicated that they perceived online interventions as a valid [24] and creditable [4] form of treatment with high levels of acceptability.

While online interventions have been shown to be effective [25-27], little is known about the degree to which the users’ engagement in, persistence with, and use of the program (or program adherence) influences the intervention outcomes. In e-therapy research trials, the term dropout is often used in place of persistence and refers to when the individual does not complete or persist with the intervention, thereby not completing the required modules or assessments [28]. However, a participant can fail to persist with the program, yet not drop out from the trial by continuing to provide outcome data. This can affect the ability of results to be generalized [29,30] and undermine the outcome of the trial. To date, efficacy trials have generally reported, when mentioned, good to excellent levels of persistence, often due to the intensive preparations, assistance, and follow-up of participants [28]. However, many authors have noted that dropout rates are high in open access trials [28,31-33], with only a small proportion persisting with the trial and associated follow-ups. For example, Christensen et al reported that only 0.5% of spontaneous users completed an open access online depression program [34], with similar figures of 1% in an open access trial for anxiety [35]. However, the dropout rates may vary according to the intervention features, with clinician-guided interventions having less dropout than interventions that are fully automated [35,36] and closed trials having better persistence (ranging from 47% to 99% [28]) than open access trials [34,37], purportedly due to the follow-up by the research team [32]. Alternatively, the low persistence rates in open access trials may be an artifact of “window shopping,” where potential users register for the program to determine what the program is like. Such an approach leads to the user completing 1 or a few modules in order to decide whether to continue with the program. The intensive preparation, informed consent, and screening processes within closed trials make such window shopping less likely, given the upfront investment and awareness in the program before commencement.

Research into program engagement, particularly the reasons for nonadherence and poor persistence, is sparse despite the thinking that the degree of engagement in self-directed or self-help online therapy is as important as engagement in face-to-face therapies [10,38,39] for optimizing outcome. What data there are indicate that users frequently cite forgetfulness and a lack of time as reasons for poor adherence [40,41] or persistence.

Maximizing a participant’s motivations to persist seems a self-evident goal of an e-therapy program [2,42], even though the moderating and mediating factors in online intervention effectiveness are not well understood [43]. Theoretical models have been developed to explain adherence and persistence on the basis of factors determined a priori by researchers in quantitative studies; drawn from medication adherence literature, health behavior change models, and technology studies; or based on the quantitative findings from online interventions. Only two studies have qualitatively explored the user perspectives and motivations that may influence adherence and persistence with asynchronous (where feedback or communication does not occur in real time but has a time delay) or automated e-therapy interventions. Gerhards et al completed a qualitative study using 18 semistructured interviews [44], concluding that three types of factors influenced adherence or persistence to, and the perceived effectiveness of, the trial: technical factors, such as knowledge and ability to access a computer; social factors, such as desire for people contact, levels of motivation, and belief that the program was applicable to the individual’s situation; and research-specific factors that may influence symptoms and symptom reporting. In a different approach, Bendelin et al classified people who participated in a guided self-help online intervention into readers, strivers, and doers based on interviews with 12 strategically selected participants [45]. Overall, motivation to persist with the intervention was influenced by participants’ perception of support they received and required, and by their perception of improvement.

While these two studies provide an understanding of some of the factors that influence adherence and the translation of online to offline behavior in their perspective populations, no clear model exists to explain the role of motivation in influencing persistence in online interventions. Grounded theory is a form of systematic qualitative inquiry that allows the generation of theory from data. This methodology is useful for the development of new theories and ideas in areas where theory does not readily exist, such as the area of motivation in online interventions and e-therapy. This study took a grounded theory approach to address the question of what influences persistence with online interventions in order to propose a model to aid program development.

## Methods

We conducted face-to-face and telephone interviews with participants in the Cardiovascular Risk E-couch Depression Outcome (CREDO) study, which evaluated the efficacy of an eHealth intervention (e-couch) in treating depressive symptoms in those with comorbid depression and cardiovascular risk factors [46].

Participants, recruited from those engaged in the study between the 6- and 12-month follow-up stages, were invited by email to participate in the qualitative study. Participants who were less than 2 weeks away from an assessment were excluded, so as not to add burden to their participation for the final survey. We sent 80 email invitations to participants with the aim of recruiting approximately 10% of these. Participants were invited only once. Participants were selected at random from those eligible using a random number generator. Recruitment occurred in three waves and continued until theoretical saturation (where no new insights emerged from the data) [47] was reached. Interviews were offered without knowledge of the study arm and were not stratified. Participants were not remunerated for their participation in either the CREDO trial or this qualitative substudy.

### Intervention

E-couch is a 12-week online cognitive behavioral therapy intervention for depression. The program contains elements pertaining to psychoeducation, activity scheduling, thought challenging, problem solving, and interpersonal therapy. The program is fully automated with a new module opening each week. During the trial, participants received a reminder email 3–4 days after the module had opened. They also received a scripted reminder, but not therapeutically assisting, telephone call from a research assistant if they had still not completed the module 1 week after it had opened.

### Ethics Approval

We obtained written informed consent from all participants. Ethics approval for the CREDO trial and the qualitative substudy was obtained from the University of Sydney Human Research Ethics Committee. The University of New South Wales Human Research Ethics Committee provided ethics approval for the 45 and Up Study.

### Study Sample

Participants in the study were residents of New South Wales, Australia, who had enrolled previously in the 45 and Up Study [48] and, subsequently, the CREDO trial [46]. Given this, all participants were aged over 45 years, had an active email address, had indicated a self-reported history or high risk of cardiovascular disease, and had at least a moderate level of depressive symptoms at two time points.

### Data Gathering

We conducted 10 telephone and two face-to-face interviews between August and December 2011 at a location and time of the interviewee’s convenience. The interviews were conducted and analyzed using a grounded theory approach, where themes and theories were allowed to emerge without using preconceived hypotheses and ideas [49]. An initial semistructured interview guide was developed with new questions being reformulated during the transcription and data analysis and added to the interview schedule in an iterative fashion. Therefore, the interview schedule evolved over the course of the research project. Interviews lasted between 46 and 68 minutes, with all interviews revolving around one key question: “What kept you using the online program after you had begun?” Theoretical saturation appeared by the ninth interview, with a further three interviews being completed to ensure that this was the case.

After we received informed consent from participants, we audio recorded interviews using an electronic dictating machine.

### Data Analysis

On completion of interviews, the interviewer transcribed audio files verbatim and then checked the transcriptions against the audio recordings. Interview transcripts were analyzed and coded as soon as possible after each interview. Handwritten field notes and impressions of the interview were also used to inform the analysis and generation of memos. The lead researcher analyzed typed transcripts of the interview using codes developed from written comments on the transcription. These comments were analyzed using a grounded theory approach emphasizing the use of iterative techniques, whereby data and emergent theory are constantly compared [49]. During the initial coding process, the lead researcher coded the transcripts to identify ideas in the data. This was followed by focused coding, where a central set of codes was pursued based on the prevalence of the initial codes and those considered a priori by the researcher. Theoretical coding was then used to link the codes to each other. Conceptual memos were written from the focused codes to help develop an understanding of the codes and how these related to the data and other codes. No coding or analysis software was necessary for this analysis.

## Results

In total, two central themes of barriers and motivators emerged, each with several subthemes. All participants reported substantial barriers that decreased their motivation to continue with the program. These included time constraints and competing priorities, technology fatigue from spending the day on the computer at work, anxiety about spending time on the computer away from other demands of day-to-day life, and perception of limited worth of the intervention.

Participants who persisted with the trial identified intrinsic motivations and extrinsically motivated strategies to overcome the barriers that they faced in persisting with the trial. These included developing habits, recognizing personal values about completion, and identifying the benefits for others if the benefits for themselves were not immediately obvious.

### Barriers

#### Personal Factors that Decreased Intrinsic Motivation to Participate

##### Forgetfulness or a Lack of Awareness That the Program Needs to be Completed

The competing demands for time and need to prioritize also appeared to result in many participants “forgetting” to complete their module and associated activities on a weekly basis. Many participants alluded to relying on weekly reminders that a module had opened, rather than initiating seeking out the new module themselves. Forgetfulness appeared to be particularly prevalent when the participants postponed completing a module until a later time. Many stated that they would put the module off, intending to complete it when they would be able to focus on the intervention without competing demands, but often forgot to return to it. They often forgot when other demands on their time arose or when they lacked regular computer time, resulting in a lack of visible reminders that the program was open.

Interviewee #11 It was good, but I did especially appreciate the reminder because sometimes it came through at a busy time, um, I didn’t mean to forget about it but it happens. And I was thankful for the reminder.

##### Mood/Anxiety

Mood and anxiety were also often cited as barriers to engagement. When anxiety was high, participants found it difficult to find the time or concentrate on the program. When mood was low, participants lacked motivation to complete the program, often feeling overwhelmed by its demands. This resulted in participants focusing on other more manageable tasks, which allowed them to feel that they had completed at least some tasks throughout the day. Tasks of daily living were often prioritized ahead of the program, even when participants believed that they would benefit from taking the time to complete the program and objectively had the time.

Interviewee #1 ...I get anxious, and then I begin to think to myself that “I’ve got to do this” and “I’ve got to do that,” and “I can’t do my [program] now, I’ll do it later,” and really there is nothing that can’t wait. Nothing at all. I have a set routine. I’m retired. But I get myself into such a state of anxiety that I can’t relax and do my [program]. So I leave it and go and do my silly little things such as taking my dog for a walk and doing my shopping. All sorts of, you know, mundane things that are not important, well they are important, but I could give myself the time to relax and do it...

#### Factors That Reduced Engagement With the Program

##### Program Seen to be a Poor Fit to the Individual

Several participants reported frustration with the program’s lack of personalization. This was particularly reinforced by frustration with the standardized questionnaires embedded in the program and trial. Participants frequently cited that the answers were unlikely to reflect their true state, as they did not believe that they fit within the categories provided. This resulted in them feeling frustrated and wanting to stop completing the questionnaires. Many indicated that the capacity to provide additional information in terms of a free-text box would be beneficial and would enhance their desire to persist with the program.

Interviewee #4 I found the questions strange. The question that said “Do you do what your doctor tells you?,” I’m going “I’m not 4,...how am I going to respond to this?” And in the end I throw my arms in despair and go “Urgh! I don’t know, I don’t know the answer!” and put it in the appropriate box...I found that when I clicked on the “Do I take x, y, z medicine” and put “Yes” and the window came up and gave me space to write in it. I want more of those windows.

Other participants were concerned that their answers were being misinterpreted, which may have influenced their answers and therefore the time taken to answer the questionnaires or complete program activities. This was reflected in the number of emails participants sent, unsolicited, to the project team to contextualize their answers to the questionnaires.

The perceived unidirectional relationship between the user and the program also led participants to consider that how they engaged in the program was irrelevant and decreased their motivation to continue. They felt that the computer offered little in terms of support, interaction, and feedback. Participants frequently reported thoughts of giving up on the questionnaires and the trial due to feeling that their information would be misrepresented and therefore not useful to the trial team.

Interviewee #1 Because you need a bit of feedback...It’s a bit of an empty system, because...you know...you put out a few stories about your thoughts and experiences, but you know...nothing comes back.

Although some participants reported that this unidirectional nature of the relationship was beneficial, for others, it reduced any obligation to log on to the system and use it. The sense of accountability was decreased, as there was no feedback or cost of engaging with the program. A lack of perception of the program being a therapeutic relationship and of someone relying on their attendance meant that several participants felt that they were able to put off completing the program. The freely available nature of the program, without cost, waiting lists, or time restrictions, meant that participants placed less value on completing the program when it was opened to them.

Interviewee #1 ...it’s a lot easier to ignore. Um...and truly and if you’ve made an appointment and you know you have to pay for it, you’re going to turn up.

##### Failure to Learn Anything New

Participants who felt they were not learning anything reported lower levels of motivation to persist with the program. Some had had previous psychological treatment for depression and felt that the program did not offer them anything substantially different.

Interviewee #1 ...think I was a bit of a difficult ah...participant, because I know a lot about uh...um...emotional therapies. And...and...a lot about the theories, so...um... it was uh...it was a bit tedious to go through each of the programs and the theories and then the examples for me...

Interviewee #2 ...a lot of the stuff we did online was not new...and that frustrated me.

Interviewee #10 Yeah, and the other thing you know, it’s long-winded. I can, sure there are some people, but for people who have, maybe, who have been to university, who have a certain level of understanding of these problems or a certain level of education um, it was too simplistic for me.

##### Lack of Perception of the Program as a Therapeutic Relationship

Previous therapy experiences also meant that some had different expectations of the therapeutic relationship. The program therefore offered reminders and information, but not therapy as such. This meant that participants struggled to engage in the computer intervention and to see it as therapy.

Interviewee #1 Um...ah...because you have no feedback, they’re like chalk and cheese really. Well, yes...it was educative, but I didn’t feel like it was a therapy session...

Those participants who perceived little personal benefit from the program reported persisting due to the perception of obligation to the researcher or the belief that their input would help the wider community. They appeared to hold values around completion and contributing to society that allowed them to continue to persist with the program.

### Motivating Factors That Enhanced Persistence and Adherence to the Program

Participants reported several strategies and motivations that allowed them to complete the program on a (semi)regular basis. These strategies were rarely linked to a specific intervention barrier but were rather seen as intrinsic traits or behaviors that allowed them to continue engaging with the program.

#### Noticing an Improvement

The perception of receiving a benefit from the program was considered to be the primary reason many participants persisted with the intervention. Participants reported experiencing benefits of the program beyond a sense of accomplishment and looking forward to completing the next part of the program. These participants frequently spoke about the benefits of the program and how they were implementing changes from the program into their daily lives.

Interviewee #3 ...if I thought to myself this is useless I’m not getting anywhere. I possibly wouldn’t finish it...I think that it was very, very interesting. There was a heck of a lot to learn from it, there was a lot I found, the reading, everything, I found I quite...I enjoyed it. There you go.

Participants who saw personal value often used the language of the program. They were able to recall tasks they completed, appeared to have a better recall of the program, and continued to refer back to the program after they had completed it. They reported an overall sense of satisfaction that carried through each module and they appeared engaged in the program as a whole.

Interviewee #5 I felt satisfied at the end of it. That I’d done it, it was interesting and it was...I suppose it reinforced what I probably should be doing myself. It was easy to do and pleasant, and yeah I felt good about doing it...

#### Feeling in Control

Several participants reported that they had dropped out of previous face-to-face therapeutic relationships due to the nature of the interactions. They reported feeling as though the therapist had been restrained by the amount of time available and that they were rushed by the time allocated to individual sessions. This resulted in their feeling unimportant and merely a patient rather than a person, and that their agenda was not as important as the agenda of the therapist.

Interviewee #3 ...you could sit there and just actually take your time to do it. You know you could really think. Whereas when you’re talking to somebody and you’ve got an hour or three-quarters of an hour or something, you really kind of you know...so time to me is the important thing.

Being able to dictate the pace in the online intervention meant that they felt they got more benefit from it. The ability to choose which activities to complete and what areas to focus on for the activities resulted in many feeling as though their agendas were catered for in the program rather than that of the therapist. Due to this, they felt that they could be more honest with the program, and this led to greater reflection.

Interviewee #11 I think when I reflect on therapy you’ve got the lead up, going to [laughs] therapy, the anxiety beforehand, you know. You’re going into the therapist’s room, you’re in someone else’s domain, but it’s like being in front of a stranger, you know, you’re in front of a stranger and you feel you have to perform a little bit. [Laughs] whereas if you’re by yourself, controlling it yourself, I think you can be more reflective about yourself.

Many participants reported returning to the intervention several times. This allowed them to reflect on what they had learned. This appeared to facilitate the program being incorporated into everyday life. Those who returned to the program several times were more likely to find the program material novel and interesting. Several of these users often frequently returned to other trusted websites when they needed information. These participants appeared to view the program as a useful tool for themselves, rather than a research project where their contribution would be more valuable to others instead of themselves.

The ability to be flexible in the completion of the program also meant that participants were able to overcome time-related barriers, allowing them to complete modules in sections, when time allowed. Sometimes this appeared to be prompted by reminders that were part of the program.

Interviewee #4 I remember doing a long one and thinking, “Oh yeah! I missed one! I can go in and do that one again”. It always good because you know you can go in and do it again. That’s the first time I actually left the memory one and come back and do it again and I’m going “I’ll do that later”

The perception of flexibility and control of the program appeared to enhance engagement in the program and therefore improved motivation to persist with the trial.

#### Sense of Duty to Oneself

Many participants cited value in completing what they had started as a reason to continue the program despite being frustrated with it. The participants often contextualized these values as being a learning of their generation, with many indicating that these values had developed as they had grown up and had persisted into adulthood.

Interviewee #2 ...because I had committed to doing something and so I did it.

Due to having these values, they were going to complete the program regardless of how frustrating or tedious it was. They completed it because their sense of what they should and shouldn’t do directed them. Holding such values meant that they did not consider stopping the program or dropping out from the study.

Interviewee #3 Sheer determination to see what’s this one going to be about, curiosity perhaps and it was determination. I feel you know if a job’s worth doing you do it as good as you can. There’s no point in giving up in the middle of [a] thing you just keep on plodding on.

On completing the entire program they described a sense of accomplishment coupled with relief, a sense of having done something that was difficult, but that they had overcome their difficulties. These participants placed value in hard work and were often scornful of those who dropped out, implying this was a weakness of character. They placed pride in completion where they saw the task as a chore rather than a personally useful tool or activity.

#### Something That Needs to be Done: Task Completion

Participants who integrated the program into their day-to-day routine appeared to be less likely to struggle to be adherent. They did not see the program as an activity to be reflected on, but rather as a task to be completed when it was available. These people did not report the frustrations with the program that those who saw completing the program due to a sense of duty did; rather, they saw it as a task to be done with little emotional connection to it.

Interviewee #2 I log in to my Internet every morning. I spend an hour or two hours, depending on what I have to do. And...um...and I’ll always complete task as they come up. So with my emails, it’s the same. I’ll answer them on the same day or possibly the next day.

This was more common in participants who logged on to the computer daily and who often referred to themselves in terms that implied a perception of being organized or task driven. Routine was often highly valued and the ability of the program to fit into this allowed it to become routine. These participants saw the program as something that needed to be done, rather than something that they wanted to do or enjoyed doing. They often described it as part of the daily chores or to-do list for that day. The development of habit and the perception of the modules as needing to be done is particularly interesting given that participants were able to opt out of the trial at any stage. Instead, they conceptualized the trial as medicine that needs to be taken.

#### Obligation to the Research and the Researchers

Many participants used words such as commitment and obligation to describe the things that made them remain engaged in the program. They described feeling a sense of commitment to the researcher or to the research. Many of these participants had been part of research teams in the past and therefore placed heavy emphasis on participating in research projects. Some were also aware of the numbers required to successfully complete a research project and aware of the impact of dropout on research outcomes.

Interviewee #9 Well, I mean, you really, you have to keep going. Um, when you start something you got to finish it, unless it drives you crazy. And it didn’t drive me crazy. There were some days when I did it and I thought “God how much more of this?,” but you made a commitment, so you followed through and you did it. And I mean if you don’t finish it how can you help if you’re only giving half information how can you help? And as I say the main criteria was perhaps I could help.

Interviewee #2 I am thinking of the fact that I have committed. Commitment is all I can think of. And I am thinking that the person I have committed to is relying on my support. And um...it would be very unfair to let them down. I know that I am only one of many people, but if everybody would drop out...where would we be?

When participants had engaged previously in programs that were not obviously linked to research, they were less likely to adhere to the program outline. This research-related engagement appeared to be driven by an obligation to others, rather than an obligation and perception of the benefits that they might have received themselves. Participants reported that when they were completing a program for only themselves, program use was more ad hoc, as they believed that they could complete the program at their own pace without affecting others. However, when engaged in research, there was more pressure to complete the program in a timely manner and in its entirety to ensure their contribution was able to benefit others.

Interviewee #2 For instance, with my [other program]. I drop in and I drop out depending on my other commitments because I know that nobody else is involved but myself.

This motivating factor appears to be linked to the completion of research and may not enhance motivation in open access users of freely available online programs. The removal of an obligation to others when there is no research project attached to the program may lead to open access users not completing programs, not obtaining the full benefits of programs, or dismissing online programs as ineffective. Such experiences may also lead to participants dismissing psychological therapies as ineffective for their problem and may actually decrease help seeking.

## Discussion

Overall, participants who had persisted with the e-therapy intervention reported multiple barriers and motivating factors in completing the program. It appeared that all participants initially engaged in the program with the hope that the program would be beneficial for themselves or for others. However, only half of the interviewed participants reported being able to complete their modules on a weekly basis. These participants appeared to have different barriers from those who completed the program less frequently, who often mentioned a lack of time and competing demands such as work and family. Motivating factors for less-adherent participants included believing that the research was important and feeling obligated to others. The more regularly adherent participants appeared to be older and reported more confusion around technology use but fewer issues with time demands and program frustrations. More-adherent participants were more likely to perceive the program as beneficial. These benefits consisted of mood improvements and a sense of getting things done, satisfaction at completing the program, and a sense of contributing to the wider community through research. However, over time the motivation to persist with the program decreased in many participants due to ongoing program frustration and the influence of personal factors such as mood issues and forgetfulness. To overcome these barriers, participants needed external motivators in the forms of reminders of their reasons for engagement and demonstrations of the benefits of completing the program (eg, changing symptom profiles). Consistent with previous research, these factors were often varied and multifaceted [42,44,45] but were incorporated into the program through the reminder emails and phone calls.

**Figure 1:**
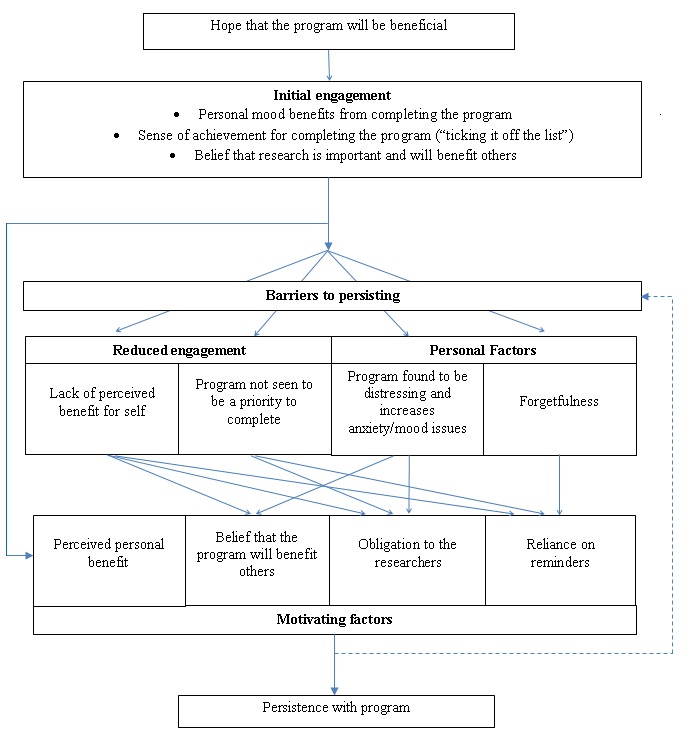
Conceptualization of the relationship between the barriers and motivating factors that influence persistence in the Cardiovascular Risk E-couch Depression Outcome (CREDO) research project.

Several factors have emerged as themes about why people choose to engage and persist in an online intervention (see Figure 1.). Consistent with the medication adherence and persistence literature [50], this study found that people appear to have to perceive a benefit of the online intervention that is greater than the cost of participating. This benefit can be personal and, in the context of a trial, altruistic. Where there were time demands and pressures to complete work, participants often appeared to report a perceived benefit of completing the program as a factor that allowed them to persist with the program. This may be the result of the perception of having completed a to-do list task and through building a sense of mastery, which may be even more important when feeling overwhelmed and pressured from less-controllable areas of their lives. This indicates the importance of highlighting achievements within the program and reinforcing participation on an ongoing basis. A sense of obligation to oneself could also be manipulated through behavioral economic approaches. For instance, patients value and perceive gaining more benefit from health interventions for which they have paid [51] and may be more likely to use these as prescribed. To our knowledge no such approach has been tried in the e-therapy arena.

However, this study suggests two additional areas that influenced persistence other than personal benefits. The first of these, which is trial specific and may not be replicated in a nontrial or real-world setting, is that participants persisted with the intervention and study because of beliefs that research is important, even when they felt little personal benefit from the program, or feelings of commitment to researchers. This is consistent with medical literature that has indicated that people who participate in research trials are more likely to view research more favorably [52] and are therefore more likely to participate. This indicates that highlighting the benefits of research in the early stages, such as recruitment, would help to initially engage people in the program and enhance their motivation to participate.

The second factor was the perception that through use of the program they may benefit the wider community. Consumers rarely appear to use interventions with the view that their behavior would affect the wider community, with the exception of vaccinations, which reduce the chance of disease for everyone through herd immunity, by decreasing exposure to pathogens. In terms of Internet-based interventions, users may be encouraged to reflect on the benefits of program completion. This might include consideration of the impact of their mood on others, the ability to provide feedback on the program so that it can be improved for others, and the importance of completing the program to determine for whom the program may be most useful, even if the participant is not finding it beneficial. While these are not necessarily trial factors per se, they may be more salient in a trial situation where participant information sheets and messages throughout the trial reinforce the experimental nature of the trial and the future benefits for others.

The influence of the research process on outcome has also been noted previously in online interventions [32,44]. Gerhards et al [44] noted that the research experience and the Hawthorne effect are likely to influence adherence and outcomes in online interventions, while Eysenbach noted that the reminders and telephone follow-up that were unique to their trial were also likely to have influenced persistence with the intervention [32]. This is consistent with our findings that reminders from the researchers influenced the participants’ persistence. However, this study also found that many participants maintained implicit psychological contracts that resulted in their feeling obligated to complete the trial due to their perception of their relationship with the researchers. Developing an understanding of the perception of this therapeutic relationship and how this can be enhanced in online trials is important for facilitating uptake [42] and use of an intervention, and enhancing persistence with the intervention once the individual is engaged [53]. This is particularly important in online interventions without clinician support, where the users may feel less obligated to the program due to the absence of a clinician or the real person at the end of the telephone or email.

Certain predicted barriers and motivational factors were absent from the themes that emerged. We expected physical discomfort and health problems to be a barrier given the older comorbid nature of the population. Many participants reported physical discomfort to the trial team through email communication, and they also often mentioned physical health problems in the interviews, but participants did not talk about these as barriers to persistence. Participants considered psychological barriers and motivators to be of greater importance than physical ones. This may reflect either that the intervention had a primary psychological target or that the Internet-based method of delivery may reduce travel-associated physical barriers. It may also represent a bias in the questions we used or in participants’ placing greater emphasis on psychological burdens and motivators. Regardless, this is consistent with other literature on uptake and motivation, which has only found psychological barriers and facilitators of persistence with interventions [42,44,45].

### Limitations

First and most important, these findings, like all qualitative research, are unique to this study population and the analysis of this research team, and the analysis of these data was influenced by the perspective of the researchers. If another team, with a different theoretical position, were to analyze the transcripts, they would likely find different results. The primary author has worked in the area of medication adherence and also works as a clinical and health psychologist, while the secondary author is a psychiatrist with a background in epidemiology. These positions have resulted in the development of views of what influences adherence behavior in a clinical setting. This, however, may be different from the influences of adherence in a research or online forum. Given this, we attempted to be as objective as possible, but the analysis is likely to have been couched within these experiences.

Second, the participants interviewed were people who volunteered to participate in a qualitative interview, from a group that volunteered to participate in an online study, from a group that volunteered to participate in a longitudinal study of health. Therefore, this group was likely to be highly motivated to participate in research and may have been different from those who did not wish to share their views. This played out in the reported emphasis of research as an important motivator by all interview participants.

Due to trial logistic constraints, participants were not contacted until approximately 9 months after they commenced the intervention, or 6 months after they had completed it. This meant that several of the participants may have struggled to recall their intrinsic motivations for continuing to engage in the trial, and their recall was likely influenced by their present situation in order to compensate for the information that they had forgotten [54]. While some of the themes that have emerged appear to be values based, and therefore quite static, it is likely the state variables such as time factors, mood, and frustration may not be as accurately reflected in these findings.

Finally, we were not able to recruit anyone who had not completed the trial or withdrawn, preventing us from exploring the barriers and motivators in people who do not persist with the intervention. Of those who did not persist and who did provide additional contact with the researchers, their reasons for dropping out of the trial tended to comprise a lack of time to complete the program at present, and they were therefore unwilling to participate in a further interview. Future research would benefit from interviewing participants who dropped out of online interventions to determine their reasons for doing so.

### Clinical Implications

In terms of maintaining participants in online programs, it may be useful to consider ways to maximize intrinsic motivations and overcome the barriers (through extrinsic motivators) that participants mentioned. This needs to occur in order to meet the dynamic state of motivation. Given this, we propose four steps.

First, briefly, initial hope could be enhanced through educating users about the benefits of Internet interventions and testimonies from previous users about the program. The use of a hook (a message or program design to build curiosity) to engage participants who are starting the intervention due to interest rather than hope for improvement may form a prelude to this and engage users early on.

Second, ongoing engagement could be enhanced through using free-text boxes and providing feedback to activities. While researchers have traditionally been cautious about free text due to the need to manage risk, participants appear to want this to enhance their experience of using the program. These aspects need to be considered if online interventions are to be used to treat people in the community without the intensive follow-up and potential obligation of a research trial.

Third, time barriers could be overcome through the use of habit-forming strategies such as having the program scheduled to be completed each week. Such scheduling would need to be balanced to ensure that program flexibility is not lost, but rather that participants could nominate a time to complete the program next week and be assigned a reminder service for this. Users could be educated about the importance of completing the program regularly and prioritizing this, in a manner similar to how they are educated about taking medication correctly. The findings from this study indicate that online interventions benefit significantly from the use of reminder systems to prompt participants who have forgotten to continue with the interventions.

Fourth, motivation could be enhanced through messaging throughout the program about the benefits of completing the program on a regular basis. Participants may benefit from personal reinforcement through having their outcome scores tracked over time and receiving tailored feedback on this. This may be particularly useful for patients who may struggle to perceive their personal improvements over the course of the program. This type of feedback needs to be provided repeatedly to keep participants motivated and to highlight their achievements and progress through the course of the program. Such an approach is similar to building mastery, which has been shown to enhance persistence with educational study [55] and with therapy [56,57]. Messaging that builds on values of completion or supporting others would also be useful. While this is somewhat difficult to generate in a public health or open access setting, the highlighting of constant monitoring and refinement of online interventions based on participant behavior and feedback may enhance program persistence for people who are completing the program for the benefit of the majority, rather than for personal gain.

However, such communication would benefit from being delivered on a variable-ratio schedule to decrease the predictability of messaging and increase interest in the communications. Further knowledge about building engagement and persistence could be drawn from the self-help book literature to further explore this area of research [58]. Finally, behavioral economic manipulation to enhance the perceived relative value of the program may also increase engagement.

This study is unique in that participants were older adults with physical health comorbidities, and little is known about the use of online interventions in such groups. In addition, the intervention consisted of 12 weekly modules, longer than the 9-module intervention studied by Gerhards et al [44] and the 8-module program used by Bendelin et al [45], thereby requiring greater persistence and motivation from the participants to complete the intervention. While the findings of this study are consistent with these earlier studies [44,45], our study proposes that persistence can be enhanced through understanding and addressing four dynamic processes: (1) building initial hope for benefits of the program, (2) enhancing personal engagement, (3) reducing barriers to use, and (4) increasing ongoing motivating factors. Future research would benefit from evaluating manipulation of the processes identified in this study to determine how these influence persistence with e-therapy interventions and the degree to which this improves outcomes.

## Abbreviations

CREDO: Cardiovascular Risk E-couch Depression Outcome
